# Advances in Lung Cancer Screening and Management: A UK–US Comparative Narrative Review Informed by American Thoracic Society (ATS) 2025 Highlights

**DOI:** 10.7759/cureus.100046

**Published:** 2025-12-25

**Authors:** Zaw Aung, Chaw Lwin Hsu, Peter Russell

**Affiliations:** 1 Respiratory Medicine, Basildon and Thurrock University Hospitals NHS Foundation Trust, Basildon, GBR; 2 Internal Medicine, East and North Hertfordshire Teaching Hospital, London, GBR; 3 Respiratory Medicine, Princess Alexandra Hospital NHS Trust, Harlow, GBR

**Keywords:** artificial intelligence, lung cancer, lung cancer screening, multidisciplinary care, radiomics, tnm staging, uk clinical practice

## Abstract

This narrative review summarises key developments presented at the American Thoracic Society (ATS) 2025 International Conference and examines their relevance to lung cancer care in the United Kingdom, with particular emphasis on transatlantic differences between US and UK practice. Drawing on selected high‑impact plenary sessions, thematic symposia, expert panel discussions, and late‑breaking abstracts covering screening, diagnosis, staging, treatment, and survivorship, the review highlights advances in lung cancer screening eligibility, emerging artificial intelligence (AI) and radiomic tools for diagnostic support, updates to Tumour-Node-Metastasis (TNM) staging, and evolving approaches to quality‑of‑life assessment. UK‑specific perspectives are incorporated, including experience from a single National Health Service (NHS) tertiary centre involving patients diagnosed with lung cancer on clinico‑radiological grounds without tissue confirmation. Differences in diagnostic strategies, healthcare system design, and resource availability between the United States and the United Kingdom are explored to identify opportunities for shared learning and adaptation within NHS lung cancer pathways. This review aims to inform UK clinicians, multidisciplinary teams, and policymakers about international developments and their potential translation into contemporary UK clinical practice.

## Introduction and background

The American Thoracic Society (ATS) International Conference is a leading global forum for emerging developments in respiratory medicine, frequently shaping international clinical practice, research priorities, and guideline development. The 2025 meeting in San Francisco featured high‑impact updates across pulmonary, critical care, and sleep medicine, with several sessions directly relevant to lung cancer screening, diagnosis, and management. While the ATS produces formal clinical practice guidelines through dedicated consensus processes, the ATS International Conference serves a different purpose, functioning primarily as a scientific forum where emerging evidence, new technologies, and evolving models of care are first shared rather than producing national, prescriptive clinical recommendations like those issued by the National Institute for Health and Care Excellence (NICE). Although hosted in the United States, ATS outputs are highly relevant to UK practice, as many innovations in screening models, diagnostic technologies, staging systems, and systemic therapies are subsequently adopted, evaluated, or adapted within the NHS.

While notable advances in systemic therapies and diagnostic techniques have been achieved, lung cancer continues to present at an advanced stage in many patients, contributing to persistently poor survival outcomes [1]. Access to early detection and diagnostic pathways remains variable across healthcare systems internationally and within the UK. In the UK, lung cancer screening is delivered via targeted lung health checks, a risk‑based screening strategy for individuals at highest risk, which has demonstrated benefits in early‑stage detection but has not yet been implemented uniformly across all NHS trusts [2]. In addition, a subset of patients are diagnosed on clinico‑radiological grounds, where imaging findings combined with the clinical context are considered sufficiently diagnostic in the absence of tissue confirmation, most often because of frailty, comorbidity, or unacceptable procedural risk. These real‑world diagnostic challenges emphasise the importance of flexible, evidence‑informed approaches to lung cancer diagnosis and management in routine clinical practice.

This narrative review synthesises selected high‑impact plenary sessions, thematic symposia, late‑breaking abstracts, and peer‑reviewed studies highlighted at ATS 2025, focusing on developments with potential relevance to UK lung cancer pathways. UK‑specific perspectives are incorporated, including survival outcomes from a retrospective cohort at a single NHS tertiary centre involving patients diagnosed with lung cancer on clinico‑radiological grounds who did not proceed to tissue biopsy. This cohort comprised predominantly advanced‑stage disease in patients deemed unfit for invasive diagnostics, reflecting real‑world multidisciplinary decision‑making in UK practice.

By integrating international advances with UK‑specific diagnostic, service-delivery, and wider healthcare system challenges, this review aims to support clinicians, multidisciplinary teams, and policymakers in translating global innovations into contemporary NHS lung cancer care.

## Review

Methods and approach

This article is a narrative review focusing on selected high‑impact content presented at the ATS International Conference 2025, with emphasis on developments relevant to UK lung cancer pathways. ATS sessions were selected based on clinical relevance, presentation format (including plenary sessions, thematic symposia, and Year in Clinical Review sessions), and their perceived translational potential for UK practice. Supporting peer‑reviewed literature was identified through targeted searches of PubMed and relevant guideline repositories, supplemented by key references cited within ATS presentations. The review prioritised recent evidence, landmark trials, and consensus statements rather than exhaustive literature capture. Formal inclusion or exclusion criteria and risk‑of‑bias assessments were not applied, consistent with a narrative review design, and the potential for selection bias is acknowledged.

Lung cancer screening: global perspectives and UK implications

US Screening Models and Equity Considerations

In the United States, lung cancer screening follows the 2021 United States Preventive Services Task Force (USPSTF) criteria: adults aged 50-80 years with a ≥20 pack-year smoking history, who currently smoke or have quit within the past 15 years [3]. However, uptake remains low - only around 18% of eligible individuals undergo screening - and concerns persist about inequity and the under-representation of high-risk minority populations [4,5].

Emerging Risk-Based Screening Approaches (ATS 2025)

At ATS 2025, several sessions highlighted emerging alternatives to the USPSTF framework, including the Life Years Gained from Screening-CT (LYFS-CT) model, which prioritises individuals based on projected life-years gained rather than risk alone. Kearney et al. reported improved sensitivity (91% vs 78%) and specificity (86% vs 84%) with these models, with particular benefits observed for Black and Hispanic populations [6].

UK Targeted Lung Health Checks (TLHC): Current Practice

In contrast, the UK is currently expanding the NHS TLHC programme, which invites adults aged 55-74 years with a history of smoking [2]. Eligibility is assessed using validated multivariable risk models such as PLCOm2012 (Prostate, Lung, Colorectal, and Ovarian Cancer risk model) and LLPv2 (Liverpool Lung Project version 2), compared to fixed criteria by the USPSTF, as summarised in Table 1. While the UK does not use a fixed pack-year threshold, individuals typically need to have smoked ≥10-20 pack-years to meet the ≥1.51% six-year risk threshold on PLCOm2012 [7].

**Table 1:** Comparison of Lung Cancer Screening Criteria in the United States and United Kingdom Comparison of lung cancer screening criteria sourced from USPSTF and UK TLHC guidance [2, 3, 7]. Abbreviations: USPSTF = United States Preventive Services Task Force; TLHC = Targeted Lung Health Check; PLCOm2012 = Prostate, Lung, Colorectal, and Ovarian Cancer risk model; LLPv2 = Liverpool Lung Project version 2.

Feature	United States (USPSTF 2021)	United Kingdom (NHS TLHC Programme)
Age range	50–80 years	55–74 years
Smoking status	≥20 pack-year history, current or quit <15 years ago	Ever-smokers (current or former)
Eligibility method	Fixed criteria	Risk prediction model (PLCOm2012 or LLPv2)
Pack-year threshold	≥20 pack-years	No fixed threshold; typically, ≥10–20 pack-years
Risk threshold	Not applicable	PLCOm2012 ≥1.51% (6-year); LLPv2 ≥2.5% (5-year)
Equity concerns	Underrepresents high-risk minority groups	Attempts to capture broader risk factors
Uptake rate	~18% nationally	Variable; expanding towards national rollout by 2029

Blood-Based Screening and Multi-Cancer Early Detection (MCED)

ATS sessions also emphasized the importance of equity and adherence in screening programmes. A key area of innovation involved blood-based pan-cancer biomarkers, such as the Galleri (GRAIL) multi-cancer early detection blood test, which uses circulating cell-free DNA (cfDNA) methylation profiling to detect multiple cancers, including lung cancer, from a single blood draw. In a large North American validation study, the Galleri test demonstrated a specificity of 99.5% for cancer signal detection, with sensitivity varying markedly by stage - ranging from 16.8% in stage I disease to 90.1% in stage IV [8]. These findings highlight important limitations in sensitivity for early-stage disease and raise concerns regarding false-negative results, as well as the potential for false-positive signals and overdiagnosis.

Importantly, the test is now being evaluated in the UK through the NHS-Galleri trial, a pragmatic randomised controlled trial enrolling over 140,000 asymptomatic individuals to assess the feasibility, clinical utility, and cost-effectiveness of this technology within the NHS screening infrastructure [9]. This reflects the UK’s growing interest in integrating minimally invasive screening tools to enhance early cancer detection and equity in population-based programmes. Blood‑based screening is easy to do and may encourage more people to take part. But before it can be used widely in the NHS, it must show clear benefits in finding cancer at an early stage, keep false‑positive results at an acceptable level, and be affordable for the health system.

Diagnosis without histology: UK experience and international reflections

A notable area of discussion was the use of clinico-radiological diagnosis in lung cancer. The UK National Lung Cancer Audit reports that approximately 18% of patients are diagnosed without tissue confirmation [1], typically due to being unfit for biopsy, declining procedures, or death occurring prior to completion of diagnostic testing. This cohort encompasses several clinical subgroups: patients presenting with advanced disease and poor functional status, those with comorbidities that render invasive procedures unsafe, and a minority who decline biopsy despite imaging findings strongly indicative of malignancy.

In a single-centre UK retrospective cohort study, overall survival (OS) among patients with a clinico-radiological diagnosis was reported to be comparable to that reported in a systematic review of untreated, histology-confirmed lung cancer (mean OS 11.2 vs 11.94 months) [10,11]. These findings were derived from a retrospective cohort study conducted at a single NHS tertiary centre, comprising predominantly patients with advanced-stage disease who were deemed unfit for invasive diagnostic procedures or declined biopsy, reflecting real-world multidisciplinary decision-making. However, this population is heterogeneous, and limited observational evidence, including single-centre retrospective analyses, suggests poorer outcomes among some younger patients and those with rapidly progressive disease.

By contrast, the United States reports lower rates of lung cancer diagnoses without histological confirmation. This likely reflects wider availability of interventional diagnostics, differing thresholds for procedural risk, and healthcare system structures that encourage more aggressive pursuit of tissue confirmation, even among frail or advanced‑stage patients. These international variations suggest that the decision to diagnose lung cancer without tissue confirmation should be guided by local healthcare structures, the availability of diagnostic resources, and prevailing clinical practice standards.

Diagnostic innovations: AI, robotics, and bronchoscopy standards

AI-Enabled Risk Prediction and Diagnostic Triage

The ATS 2025 sessions highlighted a transformative shift in lung cancer diagnostics through AI and robotic technologies. AI-driven models such as Sybil, a validated deep learning tool, have been developed to predict future lung cancer risk based on a single low-dose CT scan without the need for clinical metadata. In published validation studies, predictive C‑indices of up to 0.81 have been achieved across diverse populations, underscoring the model’s potential to scale effectively, especially in systems where specialist capacity is limited [12].

In a validation study presented at ATS, Sybil achieved a 1-year AUC of 0.86 and a 6-year AUC of 0.74 in never-smokers within a large Asian screening cohort, supporting its role in personalising screening for low-risk populations often excluded from standard eligibility criteria [13]. However, performance was attenuated in individuals with prior infectious lung changes, such as tuberculosis, highlighting the need for region-specific model refinement.

In another observational study, advanced practice providers altered management recommendations based on AI-generated Lung Cancer Prediction (LCP) scores, improving diagnostic accuracy - with the AUC increasing from 0.79 to 0.88 - and enhancing the appropriateness of referrals for invasive diagnostic procedures for suspected malignancies [14].

Robotic and Advanced Bronchoscopic Techniques

Similarly, robotic-assisted bronchoscopy is gaining prominence in the United States and has recently been introduced in multiple UK centres, including the launch at Glenfield Hospital, one of the largest cardiothoracic centres in the UK [15]. Although current NICE NG122 guidance outlines established bronchoscopic, percutaneous, and surgical diagnostic pathways, it does not yet incorporate robotic bronchoscopy, reflecting both the evolving evidence base and the usual delay between emerging innovation and formal guideline adoption. However, real-world application will depend on sustained investment in training, infrastructure, and standardisation frameworks, such as IDEAL [16] as well as careful evaluation of patient-reported outcomes. As the technology becomes more widely available, it will be essential to ensure equitable access and to integrate it effectively into existing diagnostic pathways across the NHS.

Standardising Diagnostic Yield and Procedural Quality

In the Year in Clinical Review session on lung cancer at ATS 2025, the article “Assessment of Advanced Diagnostic Bronchoscopy Outcomes for Peripheral Lung Lesions” was highlighted. This Delphi consensus, jointly developed by the American Thoracic Society and the American College of Chest Physicians, aimed to standardise the definition of diagnostic yield and improve the methodological design of future studies [17]. A 2023 meta-analysis involving over 16,000 lesions across 126 studies found no significant difference in diagnostic yield between studies conducted before and after 2012 (70.5% vs 69.2%), nor between different bronchoscopic technologies - underscoring the limitations of relying on device innovation alone without addressing study quality [18].

Robotic‑assisted bronchoscopy carries important economic and logistical implications beyond its diagnostic performance. These include high initial capital investment, ongoing maintenance requirements, longer procedural times during early adoption, and the need for structured training and multidisciplinary support. In publicly funded healthcare systems such as the NHS, these factors may shape service delivery, case selection, and overall cost‑effectiveness. This underscores the importance of robust outcome data and careful implementation planning to ensure that technological innovation translates into sustainable clinical benefit.

The panel emphasised that only specific benign or malignant diagnoses should be included when reporting diagnostic yield, excluding non-specific findings, such as inflammation or atypia. While 12-month clinical follow-up remains important, it was deemed inappropriate for yield calculation. The consensus produced 15 evidence-based statements from 19 expert panellists, advocating for the use of the IDEAL framework in study design, adherence to STARD (Standards for Reporting Diagnostic Accuracy Studies) criteria [19], and a greater focus on patient-centred outcomes. This session has refined our critical appraisal of diagnostic bronchoscopy literature and will inform both multidisciplinary team (MDT) discussions and local audits of diagnostic pathways in our clinical practice.

TNM staging: 9th edition updates

ATS 2025 provided updates on the 9th edition of Tumour-Node-Metastasis (TNM) staging. Key changes included the subdivision of N2 nodes into N2a (single station) and N2b (multiple stations), and M1c into M1c1 (multiple metastases in one organ) and M1c2 (multi-organ disease), as summarised in Appendix 1 [20]. These refinements offer greater prognostic clarity, particularly in cases of oligometastatic disease.

In UK practice, these changes are likely to inform MDT decision-making, guide patient selection for local therapies, and influence eligibility for clinical trials. The updates also highlight the importance of accurate nodal assessment (e.g., via endobronchial ultrasound [EBUS]) and raise awareness of evolving patterns in metastatic disease.

Treatment

Emerging evidence from ATS 2025 may influence treatment pathways in the UK, particularly with respect to early-stage and molecularly driven disease. Findings from the CALGB 140503 trial confirmed no significant survival difference between lobectomy, segmentectomy, and wedge resection in stage T1aN0M0 non-small cell lung cancer (NSCLC) [21], opening the door to more conservative resections where appropriate.

In the context of unresectable stage III epidermal growth factor receptor (EGFR)-positive NSCLC, the LAURA trial demonstrated a striking improvement in progression-free survival with osimertinib following chemoradiotherapy (CRT), 39.1 months versus 5.6 months with placebo [22]. This supports the case for routine EGFR testing and the use of post-CRT targeted therapy in UK practice.

For limited-stage small cell lung cancer, the ADRIATIC trial showed that adjuvant durvalumab significantly improved both overall and progression-free survival following CRT [23]. Collectively, these advances signal a shift towards personalised, biomarker-driven therapy and reinforce the importance of timely molecular testing.

Survivorship: emerging themes and QoL considerations

As lung cancer survival improves - particularly in early-stage disease and among patients receiving targeted therapies - attention is increasingly turning to survivorship and quality of life (QoL). Presentations at ATS 2025 highlighted findings from the Yale Lung Cancer Biorepository study, which used the Functional Assessment of Cancer Therapy-Lung (FACT-L) scale. The study showed that patients with early-stage NSCLC reported better longitudinal QoL than those with advanced-stage disease. It also identified several predictors of lower QoL, including younger age, low body mass index (BMI), smoking, and the presence of comorbidities [24].

These modifiable factors offer clear opportunities for targeted survivorship interventions. The UK healthcare system may benefit from routinely incorporating health-related quality of life (HR-QoL) assessments into follow-up care. In addition, embedding support for smoking cessation, physical activity, and nutritional counselling into survivorship pathways aligns with NHS ambitions to deliver holistic, personalised care.

Collectively, these insights help define priority areas for service improvement, which are outlined in Table 2 as key practice points for UK lung cancer care.

**Table 2:** Key Practice Points for UK Lung Cancer Care Abbreviations: MDT = Multidisciplinary team; TNM = Tumour, Node, Metastasis; AI = Artificial intelligence; LCP Score = Lung Cancer Prediction Score; HR-QoL = Health-related quality of life.

Key Practice Points
1. Adopt Life-Years-Gained Screening Models: Explore the future integration of life-years-gained approaches to improve equity, efficiency, and personalised eligibility in lung cancer screening programmes.
2. Strengthen Clinico-Radiological Diagnosis in MDTs: Ensure robust recognition and documentation of clinico-radiological diagnoses—particularly in frail or declining patients—so that decision-making is transparent when tissue confirmation is not feasible.
3. Implement the 9th Edition TNM Staging: Update local diagnostic and treatment pathways to incorporate the latest TNM staging revisions, supported by appropriate education and multidisciplinary training.
4. Leverage AI for Risk Prediction and Triage: Assess the utility of AI-enabled tools such as Sybil and the LCP Score to enhance risk stratification, early identification of high-risk patients, and triage in settings with limited specialist capacity.
5. Evaluate Robotic Bronchoscopy for UK Practice: Consider the potential role of robotic bronchoscopy within local services, taking into account diagnostic yield, cost-effectiveness, infrastructure, and workforce training needs.
6. Develop Structured Survivorship Pathways: Build or refine survivorship models incorporating HR-QoL assessment and targeted interventions (e.g., smoking cessation, exercise programmes, nutritional support).
7. Maintain International Engagement: Continue active participation in global scientific forums to support reflective practice and facilitate the translation of international innovations into UK lung cancer pathways.

This review also has limitations inherent to its narrative design: formal systematic searching and quantitative synthesis were not undertaken, and some evidence is drawn from conference presentations and abstracts, which may represent emerging findings not yet fully validated through peer‑reviewed publication. As the NHS Lung Cancer Screening Programme evolves, aligning these innovations with equity, access, and personalised care will be key to improving outcomes for patients across the UK.

## Conclusions

ATS 2025 highlighted major advances across the lung cancer pathway, from screening and diagnostics to treatment and survivorship. For UK clinicians, the conference reinforced the value of international collaboration and the need to adapt global evidence to local practice. Several developments appear suitable for near‑term implementation, including refinements in risk‑based screening, updates to TNM staging, improved multidisciplinary documentation for clinico‑radiological diagnosis, and greater emphasis on structured survivorship and quality‑of‑life assessment.

By contrast, innovations such as AI‑driven risk prediction tools and robotic‑assisted bronchoscopy require further validation, workforce training, infrastructure investment, and careful cost‑effectiveness evaluation before widespread adoption within the NHS. Other approaches, including blood‑based multi‑cancer detection assays, remain exploratory and are best pursued within research settings or large‑scale pilot programmes rather than routine practice.

## Appendices

Appendix 1

**Table 3:** Adapted 9th Edition TNM Classification of Lung Cancer (Proposed) Adapted from the proposed 9th Edition TNM Classification of Lung Cancer [20]. Abbreviations: TNM = Tumour, Node, Metastasis.

T/M Category	N0	N1	N2a	N2b	N3
T1a (≤1 cm)	IA1	IIA	IIB	IIIA	IIIB
T1b (>1 to ≤2 cm)	IA2	IIA	IIB	IIIA	IIIB
T1c (>2 to ≤3 cm)	IA3	IIA	IIB	IIIA	IIIB
T2a (Visceral pleura/central invasion)	IB	IIB	IIIA	IIIB	IIIB
T2a (>3 to ≤4 cm)	IB	IIB	IIIA	IIIB	IIIB
T2b (>4 to ≤5 cm)	IIA	IIB	IIIA	IIIB	IIIB
T3 (>5 to ≤7 cm)	IIB	IIIA	IIIA	IIIB	IIIC
T3 (Invasion)	IIB	IIIA	IIIA	IIIB	IIIC
T3 (Same lobe tumor nodule)	IIB	IIIA	IIIA	IIIB	IIIC
T4 (>7 cm)	IIIA	IIIA	IIIB	IIIB	IIIC
T4 (Invasion)	IIIA	IIIA	IIIB	IIIB	IIIC
T4 (Ipsilateral tumor nodule)	IIIA	IIIA	IIIB	IIIB	IIIC
M1a (Pleural/pericardial dissemination)	IVA	IVA	IVA	IVA	IVA
M1a (Contralateral tumor nodule)	IVA	IVA	IVA	IVA	IVA
M1b (Single extrathoracic lesion)	IVA	IVA	IVA	IVA	IVA
M1c1 (Multiple lesions, 1 organ system)	IVB	IVB	IVB	IVB	IVB
M1c2 (Multiple lesions, >1 organ system)	IVB	IVB	IVB	IVB	IVB
